# Manoalide Induces Intrinsic Apoptosis by Oxidative Stress and Mitochondrial Dysfunction in Human Osteosarcoma Cells

**DOI:** 10.3390/antiox12071422

**Published:** 2023-07-14

**Authors:** Zhi-Kang Yao, Yen-Hsuan Jean, Sung-Chun Lin, Yu-Cheng Lai, Nan-Fu Chen, Chung-Chih Tseng, Wu-Fu Chen, Zhi-Hong Wen, Hsiao-Mei Kuo

**Affiliations:** 1 Department of Marine Biotechnology and Resources, National Sun Yat-sen University, Kaohsiung 80424, Taiwan; akang329@vghks.gov.tw (Z.-K.Y.); d52449@auh.org.tw (Y.-C.L.); ma4949@cgmh.org.tw (W.-F.C.); 2 Department of Orthopedics, Kaohsiung Veterans General Hospital, Kaohsiung 81362, Taiwan; 3 Department of Orthopedic Surgery, Pingtung Christian Hospital, Pingtung 90059, Taiwan; jean.tang@msa.hinet.net (Y.-H.J.); linsungchun@yahoo.com.tw (S.-C.L.); 4 Department of Orthopedics, Asia University Hospital, Taichung 41354, Taiwan; 5 Division of Neurosurgery, Department of Surgery, Kaohsiung Armed Forces General Hospital, Kaohsiung 80284, Taiwan; chen06688@gmail.com; 6 Institute of Medical Science and Technology, National Sun Yat-sen University, Kaohsiung 80424, Taiwan; caviton@gmail.com; 7 Center for General Education, Cheng Shiu University, Kaohsiung 833301, Taiwan; 8 Zuoying Branch of Kaohsiung Armed Forces General Hospital, Kaohsiung 80284, Taiwan; 9 Department of Neurosurgery, Kaohsiung Chang Gung Memorial Hospital, Chang Gung University College of Medicine, Kaohsiung 833301, Taiwan

**Keywords:** osteosarcoma, inflammation, reactive oxygen species, apoptosis, antioxidants, oxidative stress, mitochondria, mitochondrial respiratory chain

## Abstract

Osteosarcoma (OS) is the most common primary malignant bone tumor that produces immature osteoid. Metastatic OS has a poor prognosis with a death rate of >70%. Manoalide is a natural sesterterpenoid isolated from marine sponges. It is a phospholipase A2 inhibitor with anti-inflammatory, analgesic, and anti-cancer properties. This study aimed to investigate the mechanism and effect of manoalide on OS cells. Our experiments showed that manoalide induced cytotoxicity in 143B and MG63 cells (human osteosarcoma). Treatment with manoalide at concentrations of 10, 20, and 40 µM for 24 and 48 h reduced MG63 cell viability to 45.13–4.40% (*p* < 0.01). Meanwhile, manoalide caused reactive oxygen species (ROS) overproduction and disrupted antioxidant proteins, activating the apoptotic proteins caspase-9/-3 and PARP (Poly (ADP-ribose) polymerase). Excessive levels of ROS in the mitochondria affected oxidative phosphorylation, ATP generation, and membrane potential (ΔΨ_m_). Additionally, manoalide down-regulated mitochondrial fusion protein and up-regulated mitochondrial fission protein, resulting in mitochondrial fragmentation and impaired function. On the contrary, a pre-treatment with n-acetyl-l-cysteine ameliorated manoalide-induced apoptosis, ROS, and antioxidant proteins in OS cells. Overall, our findings show that manoalide induces oxidative stress, mitochondrial dysfunction, and apoptosis, causing the cell death of OS cells, showing potential as an innovative alternative treatment in human OS.

## 1. Introduction

Manoalide is a natural sesterterpenoid isolated in 1980 from the marine sponge-derived West Pacific species *Luffariella variabilis* [1]. Manoalide has a variety of pharmacological activities, including antibacterial [2], calcium channel blocker [3], analgesic, anti-inflammatory [4], and anticancer properties [5,6]. Manoalide functions by permanently blocking phospholipase A2 (PLA2) with lysine residues [7]. PLA2 is a phospholipid-metabolizing enzyme that releases free fatty acids, mainly synthesizing and secreting the arachidonic acid oxidation products cyclooxygenase and lipoxygenase, which result in tumor microenvironment development, angiogenesis, and tumor growth [8]. However, the inhibition of PLA2 production affects the anticancer function [9,10]. In addition to its anti-inflammatory effects, there has not been extensive research on the anticancer effects of manoalide. It has cytotoxic effects on oral cancer, lymphoma cells, and epidermoid carcinoma cells [6]. Previous studies have found that manoalide induces apoptosis in oral cancer by the oxidative stress response [10] and DNA destruction [5]. However, studies have not yet elucidated the mechanism of mitochondrial inhibition by manoalide in osteosarcoma.

The most common bone tumor is OS [11], which is defined by mesenchymal malignant spindle cells that produce immature osteoid. OS can occur anywhere in both the appendicular and axial skeleton. However, it is most commonly found in the metaphysis of long bones and usually around the knee [12]. Three main types of treatment are used for OS: surgery, chemotherapy, and radiation therapy [13]. Patients with metastatic OS continue to have a poor long-term prognosis. At the time of diagnosis, up to 20% of OS patients will have metastatic disease [14]. Patients with metastases have survival rates of 10–40%, recurrence rates of 30–40%, and mortality >70% [15]. Despite recent attempts to enhance the effective dose and patient response in hopes of improving survival, the overall survival rate for metastatic OS has remained largely unchanged over the past 30 years [16,17]; therefore, the development and search for effective drugs are ways to improve survival.

Apoptosis, also known as programmed cell death, occurs when cells commit suicide [18]. Apoptosis is a controlled and predictable process. There are two pathways of apoptosis, one of which is the intrinsic apoptosis (mitochondria) pathway. The activation of intrinsic mitochondrial apoptosis pathway promoters and effector caspases includes poly (ADP-ribose) polymerase (PARP) and caspase-9/-3 [19]. Reactive oxygen species (ROS) are composed of a variety of oxidant molecules with different physiological effects, including superoxide anion (O_2_•−) and hydrogen peroxide (H_2_O_2_) species [20]. These are often related to oxidative stress and induce pathology via lipid, protein, and DNA damage, finally leading to apoptosis and cell death [21,22]. Cells contain antioxidants that serve as ROS scavengers to prevent apoptosis and cell damage caused by excess ROS and the reduction of oxidative stress defense enzymes [23]. Recent studies have demonstrated that exposure to high concentrations of ROS generated by chemotherapeutic drugs has cytotoxic effects and induces cancer cell apoptosis through the disruption of mitochondrial membranes and subsequent oxidative phosphorylation (OXPHOS) [24,25]. Researchers use the Seahorse XF24 extracellular flux technology and analytical instrumentation to measure and quantify changes in mitochondrial respiratory function in living cells [26,27]. The metabolism and function of mitochondria are affected by the imbalance of mitochondrial fission/fusion produced by oxidative stress, which is closely related to the occurrence and development of human diseases including neurodegenerative diseases, cardiometabolic diseases, diabetes, and cancer [28]. The purpose of this study was to investigate the mechanism by which manoalide induces oxidative stress in the OS cell line, resulting in the loss of mitochondrial function and intrinsic mitochondrial apoptosis.

## 2. Materials and Methods

### 2.1. Reagents

Manoalide and n-acetyl-l-cysteine (NAC) were purchased from Sigma–Aldrich Chemical Co. (St. Louis, MO, USA), dissolved in dimethyl sulfoxide (DMSO) and phosphate-buffered saline (PBS), protected from light, and stored at −20 °C. The FITC Annexin V Apoptosis Detection Kit was purchased from BD Bioscience (#556547, San Jose, CA, USA). The 3-(4,5-Dimethylthiazol-2-yl)-2,5-diphenyltetrazolium bromide (MTT) assay kit, MitoSOX^TM^ Red, CM-H_2_DCFDA, CellROX Green, JC-1, and DiOC6 were purchased from Thermo Fisher Scientific (Waltham, MA, USA) and dissolved in DMSO.

### 2.2. Cell Culture

MG63 cells (CRL-1427™-ATCC, Human osteosarcoma) and 143B cells (CRL-8303™- ATCC, Human osteosarcoma) were cultured with Eagle’s minimum essential medium (Gibco BRL, Rockville, MD, USA). The medium contained 10% FBS (fetal bovine serum) and glutamine–penicillin–streptomycin (2 mM–00 U/mL–100 µg/mL) (Gibco BRL). Cells were incubated under a humidified atmosphere of 5% CO_2_ room air at 37 °C. For subculture, the cells were treated with trypsin-EDTA (Gibco BRL). After centrifugation of the cells and removal of the supernatant, the cells were replanted into the dish. When the connected cells reached confluence, they had the shape of cobblestones under a microscope.

### 2.3. Cell Viability Assay

Cell proliferation (viability) was assessed using an MTT assay following treatment with different concentrations of manoalide for 24 and 48 h. MTT is a yellow substance and will interact with succinate dehydrogenase (complex II) in the electron transport chain in living cells to generate a purple substance. The cells can be lysed with DMSO to release the purple substance, and the number of living cells can be directly estimated by detecting the absorbance at 570 nm. The cells were plated in triplicate at a density of 5 × 10^3^ cells/well in 96-well plates. The cells were treated with manoalide (in 0.2% DMSO) at concentrations of 0, 0.1, 1, 5, 10, 20, and 40 μΜ for 24 and 48 h after overnight incubation. Following that, preliminary cell pattern observations were made under a phase-contrast inverted microscope (Lecia Microsystems DMI 3000B; Wetzlar, Germany). The culture solution was removed after the MTT interacted with the living cells to produce the purple substance, and 50 µL/well of DMSO was added to dissolve the purple substance fully, and the absorbance was measured at 570 nm with a spectrophotometer reader (Dynatech Laboratories, Chantilly, VA, USA). After the absorbance value of the blank group was subtracted from the absorbance values of different treatments, the following formula was used to obtain cell viability (%). Cell viability (%) = [OD570 (treatment)/OD570 (control)] × 100%. The data were expressed as the mean ± SEM.

### 2.4. Annexin V-FITC/Propidium Iodide (PI)-PE Staining

MG63 cells were treated with manoalide at the indicated concentrations of 0–10 µM for 24 h; then, the culture medium was removed, washed in PBS, trypsinized, and centrifuged, and cells were resuspended (6 × 10^5^ cells/mL) in 1× binding buffer. The samples were treated according to the manufacturer’s instructions for the FITC Annexin V Apoptosis Detection Kit (#556547, BD Biosciences, San Jose, CA, USA). Cells were first resuspended in 100 µL 1× binding buffer (6 × 10^4^ cells), and then 3 µL Annexin V-FITC and 3 µL PI-PE were added to each sample for fluorescent labeling. The samples were gently vortexed and placed at room temperature for 15 min in the dark. At the end of the incubation, 400 µL of 1× binding buffer was added to each sample, and the samples were analyzed using a CytoFLEX LX flow cytometer (Beckman-Coulter, MI, USA) with CytExpert analysis software version 2.0. We used four-quadrant flow cytometry software to detect live cells (bottom left), early apoptotic cells (bottom right), late apoptotic cells (top right), and necrotic cells (top left). At least 20,000 cells were analyzed per sample.

### 2.5. ROS Measurement

#### 2.5.1. Mitochondrial ROS

MitoSOX^TM^ Red superoxide (O_2_•−) indicators are novel fluorogenic dyes specifically targeted to mitochondria in live cells. The oxidation of the MitoSOX^TM^ Red reagent by mitochondrial ROS (mtROS) produces bright-red fluorescence. MG63 cells were treated with manoalide at 0–10 μM concentrations for 4 h and then incubated with MitoSOX^TM^ Red (5 μM) in a medium for 25 min at 37 °C, washed, trypsinized, centrifuged, and re-suspended in 1 mL of PBS. The samples were analyzed using a CytoFLEX LX flow cytometer and histograms of CytExpert analysis software. At least 20,000 cells were analyzed per sample.

#### 2.5.2. Intracellular ROS

Intracellular ROS (iROS) was evaluated by determining the level of H_2_O_2_ using the fluorescence probe chloromethyl derivative 2′,7′-dichlorofluorescin diacetate (CM-H_2_DCFD-DA), useful as an indicator of ROS in cells. This indicator exhibits much better retention in live cells than H_2_DCFDA. MG63 cells were treated with manoalide at 0, 0.1, 1, 5, and 10 μM concentrations for 4 h, incubated with 5 μM DCFH-DA in a medium for 25 min at 37 °C, washed, trypsinized, centrifuged, and re-suspended in 1 mL of PBS. The samples were analyzed using a Beckman CytoFLEX LX flow cytometer and histograms of CytExpert analysis software. At least 20,000 cells were analyzed per sample.

#### 2.5.3. CellROX^®^ Green Staining

CellROX^®^ Green reagent is a new fluorescent probe for measuring cell cytosol and nuclear oxidative stress in live cells. In six-well dishes, a density of 3 × 10^5^ cells/well was plated and left to attach overnight. After treatment with manoalide at a concentration of 0, 0.1, 1, 5, and 10 μM for 4 h, the cells were washed with PBS. The cells were then loaded with CellROX^®^ Green (5 mM) in media at 37 °C for 25 min staining, washed, trypsinized, centrifuged, and re-suspended in 1 mL of PBS. The samples were analyzed using a Beckman CytoFLEX LX flow cytometer and histograms of CytExpert analysis software. At least 20,000 cells were analyzed per sample.

### 2.6. Seahorse Real-Time Cell Metabolic Analysis

The Seahorse XF24 Extracellular Flow Analyzer (Seahorse Bioscience Inc., Chicopee, MA, USA) measures the OCR (oxygen consumption rate) and ECAR (extracellular acidification rate) in living cells, which are direct real-time quantitative indicators of mitochondrial respiration and glycolysis. For comparison between experiments, the data are expressed as OCR of pmoles/min/mg protein and ECAR of mpH/min/mg proteins. At the start, 5 × 10^4^ cells were seeded on Seahorse XF24 microplates. After overnight incubation at 37 °C, cells were treated with 0, 0.1, 1, 5, and 10 μM manoalide for 6 h. After washing the cells with 0.5 mL of Seahorse XF medium, 700 μL of Seahorse XF medium was added to each well and placed into the machine for further examination. Basal OCR was measured and plotted as a function of cells under basal conditions followed by the sequential addition of oligomycin (1 μM), carbonyl cyanide-4-(trifluoromethoxy)phenylhydrazone (FCCP; 0.5 μM), and rotenone (1 μM). At the end of the recording, cells were harvested and the amount of protein was measured using the BCA assay; then, OCR and ECAR values were calculated after normalization with the amount of protein (mg).

### 2.7. Measurement of ΔΨ_m_

#### 2.7.1. DiOC6 Staining

The cationic dye 3,3′-dihexyloxacarbocyanine iodide (DiOC6) is also a type of green fluorescent dye. It is well known as a mitochondrial membrane probe. DiOC6 can pass through the cell membrane and detect the mitochondrial membrane potential (ΔΨ_m_). In six-well dishes, a density of 3 × 10^5^ cells/well was plated and left to attach overnight. After treatment with manoalide at a concentration of 0, 0.1, 1, 5, and 10 μM for 4 h, the cells were washed with PBS. The cells were then loaded with DiOC6 (5 μM) in media at 37 °C for 20 min staining, washed, trypsinized, centrifuged, and re-suspended in 1 mL of PBS. The samples were analyzed using a Beckman CytoFLEX LX flow cytometer and histograms of CytExpert analysis software. At least 20,000 cells were analyzed per sample.

#### 2.7.2. JC-1 Kit

The positively charged mitochondrial dye JC-1 (5′,5′,6,6′-tetrachloro-1,1′,3,3′- tetraethyl benzimidazolylcarbocyanine iodide) was employed to measure ΔΨ_m_. The ΔΨ_m_ was polarized in living cells, and JC-1 will accumulate on the membrane and form a JC-1 aggregate that emits red light. The mitochondrial membrane was depolarized in dead cells. JC-1 will leave the mitochondrial membrane and enter the cytoplasm to form the JC-1 monomer and produce green light. In the six-well plate, culture medium containing 3 × 10^5^ cells and different concentrations of drugs were added for 4 h. In six-well dishes, a density of 3 × 10^5^ cells/well was plated and left to attach overnight. After treatment with manoalide at a concentration of 0, 0.1, 1, 5, and 10 μM for 4 h, the cells were washed with PBS. The cells were then loaded with JC-1 (5 μg/mL) in media at 37 °C for 20 min staining, washed, trypsinized, centrifuged, and re-suspended in 1 mL of PBS. The samples were analyzed using a Beckman CytoFLEX LX flow cytometer and four-quadrant of CytExpert analysis software. At least 20,000 cells were analyzed per sample.

### 2.8. Western Blotting

In 10 cm plates, culture medium containing 3 × 10^6^ cells and different concentrations of drugs were added for 24 h. The proteins were dissolved in a protein extraction reagent after the cells were lysed with buffer (Thermo Scientific, Waltham, MA, USA). The total protein concentration was quantified by the Bradford method (Bio-Rad, Hercules, CA, USA), and the molecular weights of the proteins in the samples differed in size, which were then separated using 8–15% SDS-PAGE electrophoresis gels, followed by transfer to PVDF (Millipore, Bedford, MA, USA) membranes. The membrane was blocked with 5% nonfat milk and then incubated overnight at 4 °C with the primary antibodies shown in Table 1. After the secondary antibody was coupled to horseradish peroxidase for 1 h at 37 °C, the signal on the membrane was detected using enhanced chemiluminescence (ECL-kit; Millipore). Photographs were taken of the visualized bands using UVP BioChemi Imaging (UVP LLC, Upland, CA, USA). The relative densitometric quantification of bands was performed using ImageJ 1.50d software (National Institutes of Health, Bethesda, MD, USA). As a loading control, the polyvinylidene fluoride membrane was re-probed with a GAPDH antibody.

### 2.9. Statistical Analysis

Data for this study were created using Microsoft Excel and plotted with GraphPad Prism 5.0 software for graphics processing. Results are expressed as the numerical mean ± standard error (SE). Student’s *t*-test was used to compare statistically significant differences between groups, where ** *p* < 0.01 or * *p* < 0.05 was considered statistically significant. Experiments were performed at least three times to verify reproducibility.

## 3. Results

### 3.1. Manoalide Significantly Induces Cytotoxicity and Apoptosis through DNA Fragmentation and Intrinsic Caspase Activation in OS Cells

**Figure 1:**
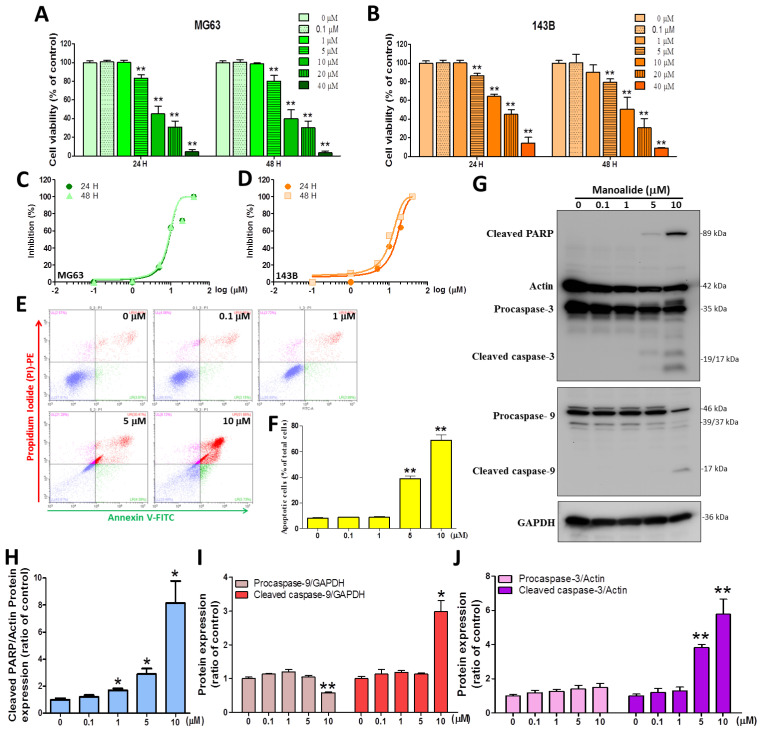
Manoalide influences cell viability, apoptosis, DNA fragmentation, and intrinsic apoptosis pathways in OS cells. (**A**) The MTT assay was used to determine the effect of manoalide. MG63 cells were treated with manoalide concentrations of 0, 0.1, 1, 5, 10, 20, and 40 µM for 24 and 48 h. Cell viability was expressed as the percentage of viable cells after drug treatment compared to untreated cells. (**B**) For 24 and 48 h, 143B cells were treated with various concentrations of manoalide. Cell viability was expressed as the percentage of viable cells after drug treatment compared to untreated cells. (**C**) Human OS cells (MG63) were treated with different concentrations of manoalide (0, 0.1, 1, 5, 10, 20, and 40 µM) for 24 and 48 h, and the drug-inhibitory ability was represented by s−curves. (**D**) Human OS cells (143B) were treated with different concentrations of manoalide (0, 0.1, 1, 5, 10, 20, and 40 µM) for 24 and 48 h and the drug-inhibitory ability was represented by s−curves. (**E**) After the treatment of MG63 with 0, 0.1, 1, 5, and 10 µM of manoalide for 24 h, the degree of apoptosis in the MG63 cells was determined using Annexin V/PI staining on a flow cytometry device. A four−quadrant dot plot created with CytExpert analysis software is shown in the figure. (**F**) With the use of CytExpert analysis software, the lower right quadrant shows that the apoptosis is in the early stage, and the upper right quadrant shows that the apoptosis is in the late stage, and the sum is produced as a bar graph. (**G**) Whole−cell lysate proteins were loaded for Western blot utilizing caspase−9, apoptosis Western blot cocktail, and GAPDH antibody after treatment with 0, 0.1, 1, 5, and 10 µM of manoalide in MG63 cells for 24 h. Blot figures were cropped from different gels, and PVDF membranes were subjected to the same conditions. Full Western blot figures are shown in Supplementary Figure S1B. The protein levels of cleaved PARP (**H**), procaspase−9/cleaved caspase−9 (**I**), and procaspase−3/cleaved caspase−3 (**J**) were quantified using ImageJ software and normalized to that of actin or GAPDH and were expressed as fold changes. Each bar represents the mean ± SE (*n* = 3) of three independent experiments, and the results were analyzed using Student’s *t*-test. * *p* < 0.05 and ** *p* < 0.01 relative to the control (0 µM manoalide).

The study used MTT reagent staining to test the cell toxicity of medical treatment with manoalide for 24 and 48 h on two OS cell lines, and the results revealed that the treatment’s effects were dose-dependent. At manoalide concentrations of 5, 10, 20, and 40 μM, the viability of the human MG63 cell line at 24 h decreased to 83.33% ± 12.38%, 45.13% ± 26.48%, 31.11% ± 20.04%, and 4.40% ± 4.78%, respectively, of that of the control (100.00% ± 6.06%, 0 μM manoalide) (Figure 1A left), while a similar effect was observed for manoalide-treated 143B cells (Figure 1B left). In MG63 and 143B cell lines, the same manoalide dose was also evaluated at 48 h (Figure 1A right and Figure 1B right). MG63 and 143B were treated with different concentrations of manoalide (0, 0.1, 1, 5, 10, 20, and 40 μM) for 24 and 48 h, and the drug inhibitory ability was measured by the MTT method and converted to s−curve data (Figure 1C,D). The IC_50_ (half-maximal inhibitory concentration) values for manoalide in MG63 cells were 8.88 ± 1.10 and 8.66 ± 1.12 μM after 24 and 48 h, respectively. The IC_50_ values for manoalide in 143B cells were 15.07 ± 1.54 and 10.93 ± 1.28 μM after 24 and 48 h, respectively (Table 2). The cytotoxic IC50 values of manoalide at 24 and 48 h were similar in MG63 and 143B cells, but at lower doses in MG63 cells, so we used MG63 cells and manoalide doses of 0, 0.1, 1, 5, and 10 μM at 4–24 h for the following experiments. Next, to elucidate the association between manoalide-induced apoptosis in OS cells, we performed an in vitro validation study. Figure 1E shows the typical four quadrants drawn between live cells (the bottom left; Annexin V-FITC^−^/PI-PE^−^), necrosis (the top left; Annexin V−FITC^−^/PI-PE^+^), early apoptotic cells (the bottom right; Annexin V−FITC^+^/PI-PE^−^), and late apoptotic cells (the top right; Annexin V−FITC^+^/PI-PE^+^) [29]. The apoptotic cells results show a dot plot shifted to the top and bottom right quadrants in MG63 cells treated with manoalide for 24 h (Figure 1E). At 5 and 10 μM manoalide, the percentage of apoptotic cells (38.80 ± 5.48% and 68.91 ± 10.96%, respectively) was significantly higher than that under 0 μM manoalide (8.22 ± 1.24%) (Figure 1F). We used the “Apoptosis Western Blot Cocktail” antibody, including procaspase−3/p17-caspase−3, cleaved PARP, and muscle actin, simultaneously detecting apoptosis-related proteins. Figure 1G shows the Western blot analysis; the treatment of MG63 cells with different dosages of manoalide for 24 h enhanced the expression levels of cleaved PARP and cleaved caspase−3/−9. However, with decreased procaspase−9 expression and unaffected procaspase−3 protein, both actin and GAPDH were used as indicators of the normalization of protein loading. MG63 cells were treated with various dosages of manoalide: 1, 5, and 10 μM, and the protein levels that cleaved PARP/actin were observed to be significantly increased to 1.69 ± 0.25-, 2.89 ± 0.69-, and 8.13 ± 2.84-fold, respectively, compared with 0 μM manoalide (1.00 ± 0.19) (Figure 1H). MG63 cells were treated with 10 μM of manoalide, and the protein levels that procaspase−9/GAPDH were observed to significantly decrease to 0.58 ± 0.04 compared with 0 μM manoalide (1.00 ± 0.08), and cleaved caspase−9/GAPDH was observed to significantly increase to 2.99 ± 0.55 compared with 0 μM manoalide (1.00 ± 0.10) (Figure 1I). MG63 cells were treated with 5 and 10 μM of manoalide, and the protein levels that cleaved caspase−3/actin were observed to significantly increase to 3.82 ± 0.31 and 5.77 ± 1.53 compared with 0 μM manoalide (1.00 ± 0.16); however, procaspase−3/actin was observed to be unaffected compared with 0 μM manoalide (Figure 1J). These data suggest that manoalide can inhibit 143B and MG63 cell viability and subsequently activate caspase−9/−3 and PARP to induce apoptosis.

### 3.2. Manoalide Treatment Increased Intracellular, Mitochondrial, and Total ROS Levels but Decreased Oxidative Stress Defense Enzyme Expression in OS

**Figure 2:**
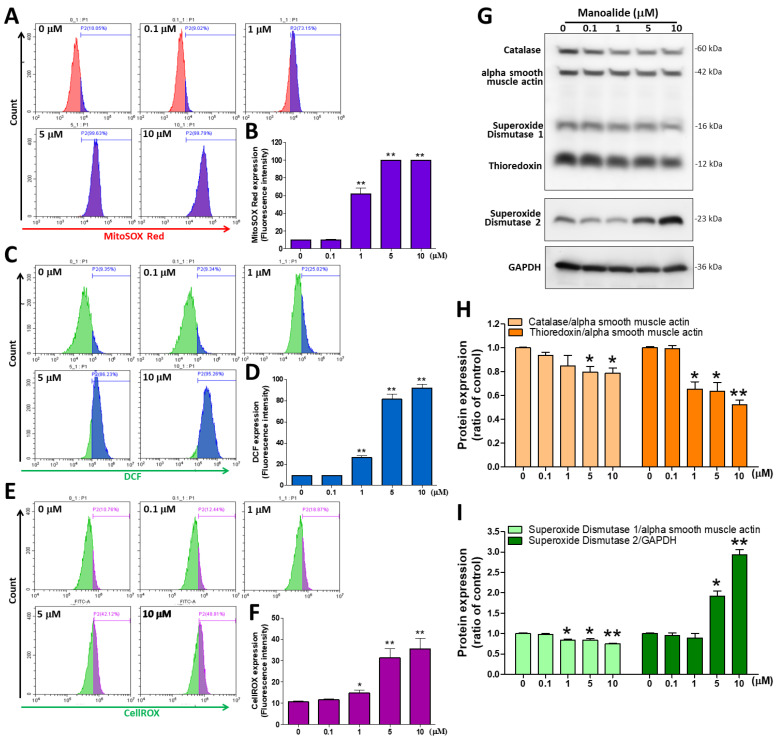
Effect of manoalide on the ROS and oxidative stress defense enzymes in MG63 cells. (**A**) MitoSOX^TM^ Red fluorescent dye was used to determine the fluorescence intensity of mitochondrial O_2_•−, which was observed by flow cytometry in the MG63 cells treated with 0, 0.1, 1, 5, and 10 µM of manoalide for 4 h. (**B**) Manoalide-induced mitochondrial O_2_•− accumulation in mitochondria was quantified by CytExpert software analysis of the gated range of 8 × 10^3^–10^6^ single-parameter histograms. (**C**) The fluorescence intensity of iROS was determined using CM-H_2_DCFDA green fluorescent dye and detected by flow cytometry in the MG63 cells treated with 0, 0.1, 1, 5, and 10 µM of manoalide for 4 h. (**D**) Manoalide-induced iROS accumulation in the cell was quantified by CytExpert software analysis of the gated range of 5 × 10^3^–10^7^ single-parameter histograms. (**E**) The fluorescence intensity of O_2_•− and •OH levels in the mitochondria and nucleus was determined using CellROX^®^ Green fluorescent dye and detected by flow cytometry in the MG63 cells treated with 0, 0.1, 1, 5, and 10 µM of manoalide for 4 h. (**F**) Manoalide-induced O_2_•− and •OH levels in the mitochondria and nucleus accumulation in the cell were quantified by CytExpert software analysis of the gated range of 6 × 10^5^–10^7^ single-parameter histograms. (**G**) In the treatment with 0, 0.1, 1, 5, and 10 µM of manoalide in MG63 cells for 24 h, whole-cell lysate proteins were loaded for Western blot by using SOD2, oxidative stress defense (catalase, SOD1, TRX, smooth muscle actin) Western blot cocktail, and GAPDH antibody. Blot figures were cropped from different gels, and PVDF membranes were subjected to the same conditions. Full Western blot figures are shown in Supplementary Figure S1C. The protein levels of catalase (**H**), thioredoxin (**H**), SOD1 (**I**), and SOD2 (**I**) were quantified using ImageJ software and normalized to that of alpha smooth muscle actin or GAPDH and were expressed as fold changes. Each bar represents the mean ± SE (*n* = 3) of three independent experiments, and the results were analyzed using Student’s *t*-test. * *p* < 0.05 and ** *p* < 0.01 relative to the control (0 µM manoalide). ROS: reactive oxygen species; iROS: intracellular ROS; H_2_DCFDA: 2′,7′-dichlorodihydrofluorescein diacetate; SOD1: superoxide dismutase 1; SOD2: superoxide dismutase 2; TRX: thioredoxin; GAPDH: glyceraldehyde-3-phosphate dehydrogenase; PVDF: polyvinylidene difluoride.

ROS are mainly produced by mitochondria, and excessive ROS production can cause oxidative stress and programmed cell death (apoptosis) [30]. Therefore, in MG63 cells treated with different dosages of manoalide, we used three ROS detection stains. The fluorescent stain probes CM-H_2_DCFDA, MitoSOX^TM^ Red, and CellROX^®^ Green were used to detect O_2_•− and •OH in the cellular components, mitochondria, and nucleus, respectively. mtROS were detected by flow cytometry using MitoSOX^TM^ Red staining; the figure shows a histogram produced using CytExpert analysis software, and we found a considerable shift to the right in MG63 cells treated with different concentrations of manoalide (Figure 2A). Based on MitoSOX^TM^ Red signals, the quantitative results indicated that mitochondrial O_2_•− levels were significantly increased in a dose-dependent manner to 61.81 ± 16.01%, 99.78 ± 0.16%, and 99.83 ± 0.05% at 1, 5, and 10 μM, respectively, in MG63 cells compared with 0 μM manoalide (9.82 ± 0.45%, Figure 2B). iROS were detected by flow cytometry using CM-H_2_DCFDA staining; the figure shows a histogram produced using CytExpert analysis software, and we found a considerable shift to the right in MG63 cells treated with different concentrations of manoalide (Figure 2C). Based on DCF fluorescent probe signals, the quantitative results indicated that intracellular hydrolytic and oxidative product levels were significantly increased in a dose-dependent manner to 26.30 ± 3.86%, 81.43 ± 9.26%, and 91.82 ± 7.06% at 1, 5, and 10 μM for MG63 cells, respectively, compared with 0 μM manoalide (9.30 ± 0.54%, Figure 2D). Similarly, O_2_•− and •OH levels in the mitochondria and nucleus were detected by flow cytometry using CellROX^®^ staining; the figure shows a histogram produced using CytExpert analysis software, and we found a considerable shift to the right in MG63 cells treated with different concentrations of manoalide (Figure 2E). The quantitative results indicated that O_2_•− and •OH levels were significantly increased in a dose-dependent manner, based on CellROX^®^ Green probe signals, to 14.77 ± 3.56%, 31.34 ± 10.39%, and 35.59 ± 12.17% at 1, 5, and 10 μM, respectively, in MG63 cells compared with 0 μM manoalide (10.59 ± 1.11%, Figure 2F). We used the “human oxidative stress defense enzymes Western blot cocktail” antibody containing catalase, superoxide dismutase 1, thioredoxin, and alpha smooth muscle actin, which are involved in protecting cells against oxidative stress and the regulation of ROS activity. Superoxide dismutase 2 (SOD2; Mn-SOD) is situated in the mitochondrial matrix to scavenge ROS and avoid superabundance of mtROS production and prevent oxidative stress [31]. Figure 2G shows the Western blot analysis revealed that treating MG63 cells with various dosages of manoalide for 24 h increased the expression level of SOD2 but decreased the expression of catalase, superoxide dismutase 2 (Cu-Zn SOD, SOD1), and thioredoxin (TRX) proteins, with GAPDH and alpha smooth muscle actin serving as indicators of the normalization of protein loading. Manoalide was applied to MG 63 cells at concentrations of 5 and 10 M for 24 h, and the protein levels of catalase/alpha smooth muscle actin were significantly decreased to 0.80 ± 0.08 and 0.79 ± 0.08, respectively, compared with 0 μM manoalide (1.00 ± 0.01), and the protein expression of TRX/alpha smooth muscle actin was significantly decreased to 0.65 ± 0.11, 0.64 ± 0.13, and 0.52 ± 0.07, respectively, compared with 0 μM manoalide (1.00 ± 0.02) (Figure 2H). We also observed that the protein levels of SOD1/alpha smooth muscle actin were significantly reduced to 0.84 ± 0.03, 0.84 ± 0.05, and 0.75 ± 0.03, respectively, compared with 0 μM manoalide (1.00 ± 0.04), tested at concentrations of 1, 5, and 10 M in MG63 cells. However, in MG63 cells treated with 5 and 10 μM manoalide, a significant increase in SOD2/GAPDH expression was observed at 1.92 ± 0.21 and 2.94 ± 0.21, respectively, compared with 0 μM manoalide (1.00 ± 0.04) (Figure 2I). Taken together, manoalide induced intracellular, mitochondrial, and nuclear ROS overproduction in OA cells while unbalancing the activity of antioxidant enzymes, causing oxidative stress and contributing to cell apoptosis.

### 3.3. Manoalide Treatment Reduces OCR and Oxidative Phosphorylation (OXPHOS) Protein Expression in MG63 Cells

**Figure 3:**
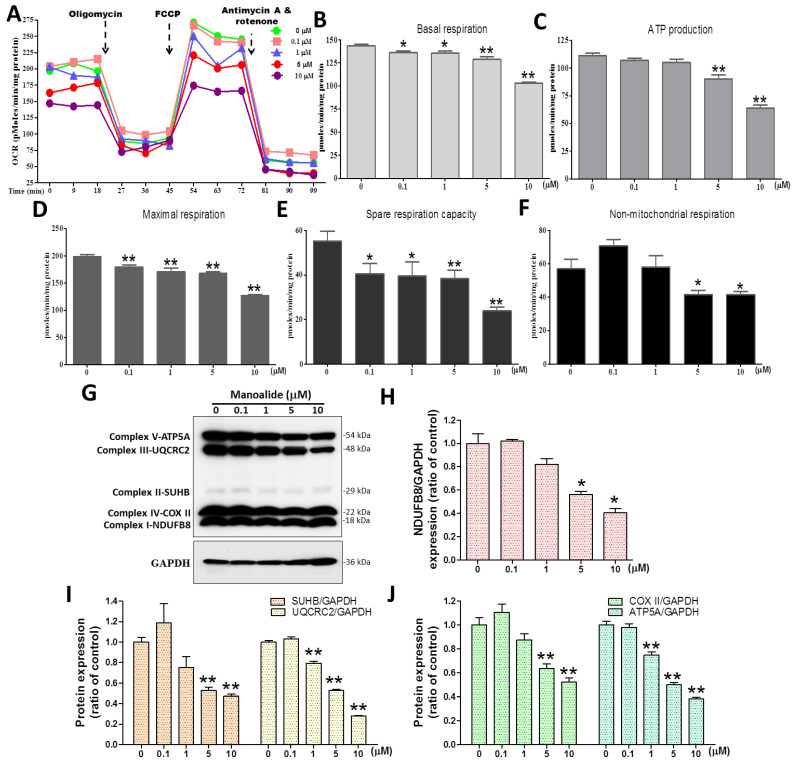
Effect of manoalide on the oxygen consumption rate and OXPHOS enzymatic complex protein in MG63 cells. (**A**) OCR values (pmoles/min/mg protein) and time (minutes) graphs were measured after MG63 cells were treated with various concentrations of manoalide for 4 h, followed by continuous injection of Seahorse XF Cell Mito Stress kit reagents, including oligomycin, FCCP, and antimycin/rotenone. In MG63 cells treated with 0, 0.1, 1, 5, and 10 μM manoalide, the parameters quantified and analyzed were (**B**) basal respiratory capacity, (**C**) ATP production (coupled respiration), (**D**) maximal respiratory capacity, (**E**) spare respiratory capacity, and (**F**) non-mitochondrial respiratory capacity. These data were quantified by normalizing cellular protein concentrations. (**G**) In the treatments with 0, 0.1, 1, 5, and 10 µM of manoalide in MG63 cells for 24 h, whole-cell lysate proteins were loaded for Western blot by using total OXPHOS human WB antibody cocktail and GAPDH antibody. Blot figures were cropped from different gels, and PVDF membranes were subjected to the same conditions. Full Western blot figures are shown in Supplementary Figure S2A. The protein levels of NDUFB8 (**H**), SDHB (**I**), UQCRC2 (**I**), COX II (**J**), and ATP5A (**J**) were quantified using ImageJ software and normalized to that of GAPDH and were expressed as fold changes. Each bar represents the mean ± SE (*n* = 3) of three independent experiments, and the results were analyzed using Student’s *t*-test. * *p* < 0.05 and ** *p* < 0.01 relative to the control (0 µM manoalide). OXPHOS: oxidative phosphorylation; OCR: oxygen consumption rate; FCCP: *Carbonyl cyanide*-*4-(trifluoromethoxy*)*phenylhydrazone*; WB: Western blotting; GAPDH: glyceraldehyde-3-phosphate dehydrogenase; PVDF: polyvinylidene difluoride; NDUFB8: NADH dehydrogenase [ubiquinone] 1 beta subcomplex subunit 8; SDHB: succinate dehydrogenase complex iron sulfur subunit B; UQCRC2: ubiquinol–cytochrome C reductase core protein 2; COX II: cytochrome c oxidase subunit II; ATP5A: ATP synthase subunit alpha.

The Seahorse XF24 extracellular flux bioenergy metabolism analyzer, developed by Seahorse Bioscience in the United States, is the only platform in the world that can evaluate the overall energy metabolism of living samples. To complete the mitochondrial function test, the Cell Mito Stress Test kit was used to first detect the basic oxygen consumption; then, an ATP synthase inhibitor (oligomycin) was added to inhibit the mitochondria’s production of ATP, and the inhibited oxygen consumption indicates how much oxygen is involved in the synthesis of ATP. Then, at the appropriate concentration, FCCP, an uncoupler medication, was added without disrupting the electron transport chain and enabling the mitochondria to remain idle under harsh conditions to determine the mitochondria’s maximum oxygen consumption capacity. Finally, Complex I, an electron transport chain inhibitor, was added. The background value for its detection is determined by the inhibitor rotenone and the complex III inhibitor Antimycin A, which fully shuts down mitochondrial oxygen utilization. As a result, the following mitochondrial respiration parameters can be calculated: basal respiration, ATP-linked production (coupled respiration), maximal respiration, spare respiratory capacity, and non-mitochondrial respiration. MG63 cells were treated with various dosages of manoalide, followed by sequential addition of oligomycin, FCCP, and rotenone/actinomycin, which were found to decrease OCR parameters (Figure 3A). With the increase in manoalide concentration in MG63 cells, the mitochondrial basal respiratory values decreased significantly to 136.12 ± 5.40, 135.38 ± 7.66, 129.07 ± 7.28, and 102.97 ± 4.31 pmoles/min/mg protein at 0.1, 1, 5, and 10 μM compared with the 0 μM manoalide group (143.43 ± 6.09) (Figure 3B). With the increase in manoalide concentration in MG63 cells, the mitochondrial ATP production values decreased significantly to 90.29 ± 10.33 and 63.71 ± 8.13 pmoles/min/mg protein at 5 and 10 μM compared with the 0 μM manoalide group (111.00 ± 6.94) (Figure 3C). With the increase in manoalide concentration in MG63 cells, the mitochondrial maximal respiration values decreased significantly to 179.11 ± 13.15, 170.74 ± 20.08, 167.48 ± 9.43, and 126.85 ± 5.83 pmoles/min/mg protein at 0.1, 1, 5, and 10 μM manoalide concentrations compared with the 0 μM manoalide group (198.63 ± 12.13) (Figure 3D). With the increase in manoalide concentration in MG63 cells, the mitochondrial spare respiration capacity values decreased significantly to 40.46 ± 13.87, 39.56 ± 18.12, 38.42 ± 11.16, and 23.88 ± 4.82 pmoles/min/mg protein at 0.1, 1, 5, and 10 μM manoalide concentrations, compared with the 0 μM manoalide group (55.20 ± 13.32) (Figure 3E). With the increase in manoalide concentration in MG63 cells, the nonmitochondrial respiration values decreased significantly to 41.66 ± 7.86 and 41.56 ± 7.64 pmoles/min/mg protein at 5 and 10 μM manoalide concentrations compared with the 0 μM manoalide group (57.03 ± 19.23) (Figure 3F). We used the “Total OXPHOS Human WB Antibody Cocktail” antibody including complex I–V to detect five OXPHOS complex-related proteins. Figure 3G shows in the Western blot analysis that the application of MG63 cells with various dosages of manoalide for 24 h decreased OXPHOS complex I–V protein expression, with GAPDH used as an indicator of normalization of protein loading. Manoalide was applied to MG 63 cells at concentrations of 5 and 10 μM, and the protein levels of the complex I-NDUFB8/GAPDH were observed to be significantly decreased to 0.56 ± 0.04 and 0.41 ± 0.06, respectively, compared with 0 μM manoalide (1.00 ± 0.14) (Figure 3H). Manoalide was applied to MG 63 cells at concentrations of 5 and 10 μM, and the protein expression of the complex II-SUHB/GAPDH was observed to be significantly decreased to 0.53 ± 0.05 and 0.47 ± 0.03, respectively, compared with 0 μM manoalide (1.00 ± 0.08); however, the protein expression of the complex III-UQCRC2/GAPDH was observed to be significantly decreased to 0.79 ± 0.03, 0.53 ± 0.02, and 0.28 ± 0.01 at 1, 5, and 10 μM, respectively, compared with 0 μM manoalide (1.00 ± 0.02) (Figure 3I). Manoalide was applied to MG 63 cells at concentrations of 5 and 10 μM, and the protein expression of the complex IV-COX II/GAPDH was observed to be significantly decreased to 0.63 ± 0.07 and 0.52 ± 0.06, respectively, compared with 0 μM manoalide (1.00 ± 0.10). However, the protein expression of the complex V-ATP5A/GAPDH was observed to be significantly decreased to 0.75 ± 0.04, 0.50 ± 0.03, and 0.38 ± 0.02 at 1, 5, and 10 μM, respectively, compared with 0 μM manoalide (1.00 ± 0.05) (Figure 3I). These findings suggest that manoalide effectively decreased mitochondrial respiration function and OXPHOS complex I–V protein expression, causing a loss of mitochondrial function in MG63 cells.

### 3.4. In MG63 Cells, Manoalide Regulates Mitochondrial Transmembrane Potential (ΔΨ_m_) and Mitochondrial Dynamic Protein

**Figure 4:**
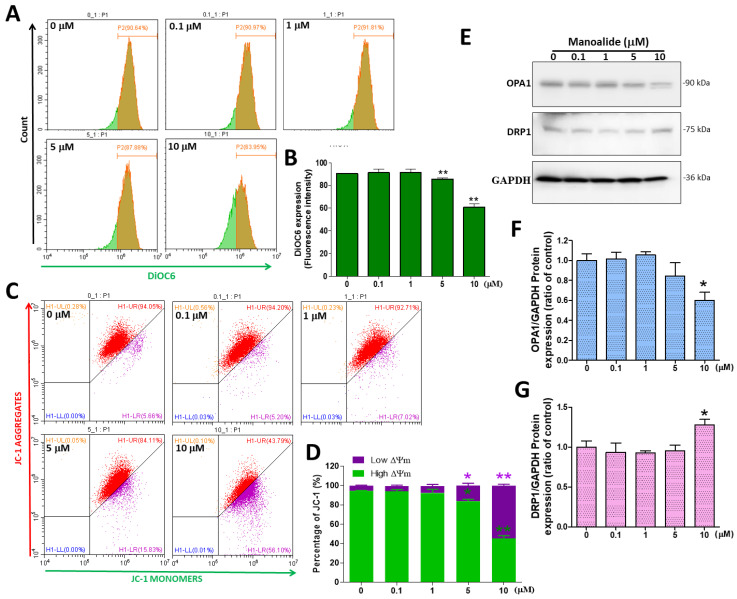
Effects of manoalide on ΔΨ_m_ and the expression levels of mitochondrial dynamic proteins in MG63 cells. (**A**) The fluorescence intensity of ΔΨ_m_ in MG63 cells treated with 0, 0.1, 1, 5, and 10 µM of manoalide after 4 h was evaluated using DiOC6 green fluorescent dye and identified by flow cytometry. (**B**) The manoalide-induced reduction in ΔΨ_m_ was quantified by CytExpert software analysis of the gated range of 8 × 10^5^–10^7^ single-parameter histograms. (**C**) After treatment with 0, 0.1, 1, 5, and 10 µM of manoalide for 4 h in MG63 cells, JC-1 labeling by flow cytometry revealed a decrease in red fluorescence, indicating mitochondrial depolarization. A four-quadrant dot plot created with CytExpert analysis software is shown in the figure. (**D**) Through analysis of the selected range of the quadrant plot, CytExpert quantitated JC-1 signals. The aggregated and monomer JC-1 amount was determined using the high and low ΔΨ_m_ (upper right and lower right) quadrants. (**E**) The treatments with 0, 0.1, 1, 5, and 10 µM of manoalide in MG63 cells for 24 h: whole-cell lysate proteins were loaded for Western blot by using OPA1, DRP1, and GAPDH antibodies. Blot figures were cropped from different gels, and PVDF membranes were subjected to the same conditions. Full Western blot figures are shown in Supplementary Figure S2B. The protein levels of OPA1 (**F**) and DRP1 (**G**) were quantified using ImageJ software and normalized to that of GAPDH and were expressed as fold changes. Each bar represents the mean ± SE (*n* = 3) of three independent experiments, and the results were analyzed using Student’s *t*-test. * *p* < 0.05 and ** *p* < 0.01 relative to the control (0 µM manoalide). DiOC6: 3,3′-dihexyloxacarbocyanine iodide; ΔΨ_m_: mitochondrial membrane potential; JC-1: 5′,5′,6,6′-tetrachloro-1,1′,3,3′-tetraethylbenzimidazolyl-carbocyanine iodide; OPA1: optic atrophy 1; DRP1: dynamin-related protein 1; GAPDH; glyceraldehyde-3-phosphate dehydrogenase; PVDF: polyvinylidene difluoride.

Although mitochondria are the source of ROS, excessive ROS generation may be the cause of oxidative stress and cell death, followed by ΔΨ_m_ loss and mitochondrial dynamic imbalance [32]. Several lipophilic cationic fluorescent dyes, such as DiOC6 and JC-1 (37 °C, 20 min), bind to the mitochondrial matrix in live eukaryotic cells, and the amplification or weakening of their fluorescence suggests an enhancement or decrease in mitochondrial internal membrane electronegativity. ΔΨ_m_ was detected by flow cytometry using a DiOC6 probe; the figure shows a histogram created using CytExpert analysis software, and we observed a significant shift to the left in MG63 cells treated with various doses of manoalide (Figure 4A). Based on DiOC6 signals, the quantitative results indicated that ΔΨ_m_ levels were significantly decreased in a dose-dependent manner, to 85.61% ± 1.97% and 60.83% ± 5.02% at 5 and 10 μM in MG63 cells, respectively, as compared to controls (90.62% ± 0.15%, Figure 4B). In healthy cells, the JC-1 dye accumulates on the inner membrane of the mitochondria, forming the aggregate that emits red light. When apoptosis occurs, the membrane potential of the mitochondria decreases and the dye returns to the cytoplasm, comprising monomer and emitting green light [33]. Figure 4C shows the typical four-quadrant diagram in which findings reveal a dot plot that moved from the right upper quadrant to the right bottom quadrant in MG63 cells treated with manoalide for 4 h. The percentage of low ΔΨ_m_ (16.09 ± 4.25% and 54.37 ± 2.56%, respectively) at 5 and 10 μM manoalide was considerably greater than that in the 0 μM manoalide group (5.37 ± 0.71%), but the percentage of high ΔΨ_m_ (83.87 ± 3.35% and 45.58 ± 4.70%, respectively) was substantially lower than that in the 0 M manoalide group (94.58 ± 0.74%) (Figure 4D). Mitochondrial fission and fusion are involved in mitochondrial quality control and transfer of energy state. The increased production of OXPHOS and ATP during mitochondrial fusion may induce mitochondrial fusion protein to prevent cell death. In contrast, mitochondrial fission leads to ATP depletion and OXPHOS deficiency, causing apoptosis [34]. Figure 4E shows the Western blot where the treatment of MG63 cells with various dosages of manoalide for 24 h increased the expression levels of fission-associated protein DRP1 but decreased the expression level of fusion-related proteins OPA1, with GAPDH used as an indicator for the normalization of protein loading. As MG63 cells were treated with 10 μM manoalide, the protein levels of OPA1/GAPDH were found to be considerably lower, at 0.60 ± 0.14, when compared to the control (1.00 ± 0.11, 0 μM manoalide) (Figure 4F). When MG63 cells were treated with 10 μM manoalide, the protein expression of DRP1/GAPDH increased significantly to 1.28 ± 0.12 compared to the control (1.00 ± 0.14, 0 μM manoalide) (Figure 4G). These findings demonstrated that utilizing varying doses of manoalide reduced high ΔΨ_m_ and increased mitochondrial fission protein but reduced the fusion proteins in MG63 cells, resulting in cell death.

### 3.5. N-Acetylcysteine Pre-Treatment Reduces Manoalide-Induced Apoptosis, Cellular ROS Production, and Oxidative Stress Defense Enzyme Expression

**Figure 5:**
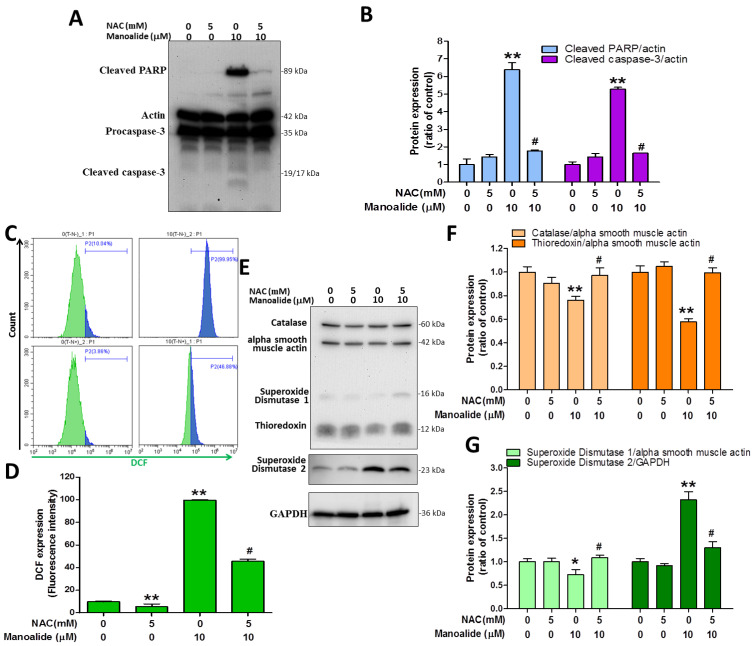
Pretreatment with antioxidant NAC partially rescued apoptosis, iROS accumulation, and antioxidant enzymatic protein induced by manoalide in MG63 cells. (**A**) Using the “Apoptosis Western Blot Cocktail” antibody, Western blot analyses of cells treated with or without NAC and manoalide were performed. Blot figures were cropped from different gels, and PVD membranes were subjected to the same conditions. Full Western blot figures are shown in Supplementary Figure S2C. The protein levels of cleaved PARP and procaspase-3 (**B**) were quantified using ImageJ software and expressed as fold changes when adjusted to actin levels. (**C**) The fluorescence intensity of iROS in MG63 cells treated with or without NAC and manoalide for 24 h was evaluated using CM-H_2_DCFDA green fluorescent dye and identified by flow cytometry. (**D**) Quantitation of the DCF detection signals by CytExpert software analysis of the gated range of 5 × 10^3^–10^7^ single-parameter histograms. (**E**) Western blot analyses of cells treated with or without NAC and manoalide using Oxidative Stress Defense (catalase, SOD1, TRX, smooth muscle actin) Western Blot Cocktail, SOD2, and GAPDH antibody. Blot figures were cropped from different gels, and PVD membranes were subjected to the same conditions. Full Western blot figures are shown in Supplementary Figure S2D. The protein levels of catalase (**F**), TRX (**F**), SOD1, and SOD2 (**G**) were quantified using ImageJ software and normalized to that of actin, and GAPDH was expressed as the fold change. Each bar represents the mean ± SE (*n* = 3) of three independent experiments, and the results were analyzed using ANOVA. * *p* < 0.05, **; *p* < 0.01 relative to the control group (without NAC and manoalide), and ^#^ *p* < 0.05 relative to the experimental group with 10 μM of manoalide alone. iROS: intracellular ROS; NAC: N-acetylcysteine; PARP: poly(ADP-ribose) polymerase; SOD1: superoxide dismutase 1, TRX: thioredoxin.

N-acetylcysteine (NAC) is a reducing agent that functions as an antioxidant by depleting ROS in cells [35]. MG63 cells were or were not exposed to 5 mM NAC for 2 h to determine the effects of NAC on manoalide-induced cellular ROS overproduction, the reduction of oxidative stress defense enzymes, and apoptosis. After that, 10 μM manoalide was administered and allowed to respond for 24 h to evaluate immunoblot expression levels of cleaved PARP and cleaved caspases 3 from MG63 cells treated with or without NAC and 10 µM manoalide. The results demonstrated that manoalide dramatically enhanced the expression levels of cleaved caspase 3 and cleaved PARP, whereas NAC treatment reversed this and decreased the levels (Figure 5A,B). We pretreated MG63 cells for 2 h with or without 5 mM NAC and then performed studies with or without 10 µM manoalide for 4 h in the incubator, followed by staining MG63 cells with CM-H_2_DCFDA dye and analyzing flow cytometry. The findings showed that NAC did not generate iROS and that iROS levels were strongly increased following manoalide therapy, which was substantially reduced by NAC pre-treatment (Figure 5C,D). To evaluate the immunoblot expression levels of catalase, TRX, SOD1, and SOD2 proteins, MG63 cells were administered 10 μM manoalide and 5 mM NAC. The results showed that manoalide dramatically reduced the protein expressions of catalase, TRX, and SOD1; this was reversed when NAC was administered. Meanwhile, it was observed that NAC did not cause SOD2 protein changes, and SOD2 protein was significantly elevated after manoalide treatment, which was partially restored by NAC pre-treatment. (Figure 5E–G). These findings show that NAC dramatically reverses apoptotic protein, ROS production, and oxidative stress defense enzyme expression generated by manoalide, confirming ROS as the primary underlying mechanism causing the aforementioned effects.

## 4. Discussion

OS is the most frequent primary bone tumor [11], resulting from malignant mesenchymal spindle cells that produce immature osteoid [36]. Surgery, chemotherapy medicines, and radiation therapy are the three major treatments for OS [14]. Patients with metastatic OS continue to have a terrible prognosis, with only a 10–40% survival rate and >70% mortality [15]. Therefore, one strategy to improve survival is to research or develop new drugs. In the last ten years, there has been significant growth in the number of biologically active medications for cancer therapy and prevention, and manoalide is one of them. Manoalide is a natural sesterterpenoid, a marine medicine obtained from sponges, whose structure is shown in Supplementary Figure S1A [4]. Calcium channel blockers [3] and phospholipase A2 (PLA2) inhibitors [37] are two known modes of action for manoalide. PLA2 is a phospholipid-metabolizing enzyme that mainly synthesizes and secretes arachidonic acid oxidation products from cyclooxygenase and lipoxygenase, contributing to tumor microenvironment development, angiogenesis formation, and tumor growth. Apart from its anti-inflammatory effects, the anticancer effects of manoalide have not been extensively studied. It is only cytotoxic to oral cancer [10], human squamous cell carcinoma [3], and epidermoid cancer cells [6], induced by oxidative stress [10], apoptosis, and DNA deterioration to oral cancer [5]. The treatment and molecular mechanisms of action of manoalide in OS have not been studied. Our experimental results showed that manoalide exhibited the most potent inhibitory effect on the proliferation of MG63 and 143B cells, and low doses disrupted cell growth with IC_50_ of approximately 8.7 versus 10.9 µM for 48 h. Manoalide has been reported to have antitumor activity in oral cancer studies with an IC_50_ of approximately 14.0 μM for 48 h, similar to our experiments [10]. However, we found that the IC_50_ of MG63 cells was approximately 8.9 µM for 24 h, with very little difference from 48 h. Our study found that the difference in manoalide had a distinct typical anti-viability effect on human OS cancer cells.

Most newly manufactured chemicals are thought to have complex mechanisms that promote apoptosis, and targeting apoptosis signaling is developing as a method for novel cancer therapies [38,39,40]. The caspase family of apoptosis is typically classified into two categories: intrinsic and extrinsic activators, of which the intrinsic activation pathway belongs to the mitochondrial pathway including caspase-9/-3. Caspases’ most important function in cells is to operate as a catalytic inactivator of genes, which requires proteolytic activation during apoptosis, and N-terminal peptides have no similarity, and once caspases are activated, most cellular targets are proteolytically cleaved by effector caspases, which results in cell death [41]. Boulares et al. (1999) demonstrated that apoptosis in the cell requires the immediate interruption of nucleoprotein poly(ADP-ribosyl)ation, accompanied by cleavage by caspase-3 catalyzed PARP; PARP is then cleaved into fragments of 89 and 24 KDa, enclosing the enzymatic activity and the DNA-binding domain [42]. Our study of manoalide showed that anticancer activity occurs through the intrinsic apoptotic pathway. Annexin V/PI staining of cells revealed quantitative early and late apoptotic bodies, and the cleaved forms of caspase-9/-3 and PARP were activated. Thus, our study shows that manoalide induces apoptosis by activating caspase-9/-3 and PARP cleavage in an intrinsic manner.

Oxidative stress is a biochemical situation defined by the presence of relatively large amounts of harmful reactive species, primarily made up of ROS, and an imbalance between antioxidant defense mechanisms. ROS are primarily produced in cells as byproducts of regular mitochondrial metabolism and have long been linked to apoptosis induction [43,44]. NAC is an aminothiol that acts as an intracellular precursor for the synthesis of cysteine and glutathione, making it a significant antioxidant. NAC has been frequently employed as a research tool in the field of apoptosis research to investigate the role of ROS in apoptosis induction. Manoalide triggers the overproduction of mtROS, iROS, and nROS, affecting the reduction of intracellular antioxidant enzyme proteins (oxidative stress defense enzymes: catalase, SOD1, and TRX), but the only increase is the mitochondrial antioxidant enzyme SOD2. SOD2 will convert mitochondrial superoxide O^2−^ to H_2_O_2_, and then the antioxidant enzyme protein (TRX) that removes H_2_O_2_ is converted into nontoxic H_2_O because TRX is decreased and it is too late to remove ROS, and ROS are sent to the cytoplasm, resulting in a large increase in intracellular ROS, which induces oxidative stress. The iROS can destroy proteins and DNA to induce pathology, leading to apoptotic cell death. Oral cancer studies showed that manoalide increases ROS [5,10], but there is no proof that antioxidant enzyme proteins change, and we are the first to find that manoalide-induced antioxidant enzyme protein (oxidative stress defense enzymes: catalase, SOD1, and TRX) decreased and the mitochondrial antioxidant enzyme SOD2 increased in OS cells. Therefore, we know that manoalide can affect oxidative stress to cause ROS accumulation and inhibit antioxidant enzyme protein, but the increase in SOD2 can also cause a large amount of ROS to be generated, and the double addition can lead to apoptosis, finally resulting in cell death.

Mitochondria play an important role in eukaryotic cells, where their function is to generate ATP during OXPHOS. Studies have shown that manoalide reduces nonmitochondrial (in the cytoplasm) and OXPHOS (in the mitochondria) respiration, including basal respiratory capacity, ATP production, maximal respiratory capacity, spare respiratory capacity, and nonmitochondrial respiratory. The inner mitochondrial membrane has several folds, among which are components of the respiratory chain or OXPHOS complexes I to V. Complexes I to V are multi-subunit enzymes that can synergistically generate an electrochemical proton gradient on the inner mitochondrial membrane. According to research, manoalide reduces the total number of OXPHOS complex I to V proteins, which combined with complex V (ATP synthase) form the mechanism for ATP generation [45]. It is worth emphasizing that mitochondrial malfunction occurs before ΔΨ_m_ damage, nuclear condensation, and the generation of apoptotic bodies [46]. Studies have shown that the potent cytotoxicity and induction of apoptosis caused by manoalide in OS cells are achieved through the induction of mtROS, mitochondrial dysfunction, and the destruction of ΔΨ_m_. Mitochondria are active organelles that perform fusion (combination of fragments) and fission (splitting into small fragments). The inner membrane protein OPA1 is required for mitochondrial fusion, and the DRP1 protein is required for mitochondrial fission. For rapid and efficient apoptosis, mitochondria must be expressed in fragments through a highly permeable outer surface, and cristae should be separated for controlling mitochondrial morphology and not allowing content exchange between mitochondria [47]. As a result, mitochondrial fission is critical for the response to oxidative stress and apoptosis [48]. Our findings support the previous assessment that manoalide-induced apoptosis is responsible for the reduction in mitochondrial fusion protein expression and the rise in mitochondrial fission protein expression in OS cells. Although not all cells or signaling pathways are linked to apoptosis and mitochondria, many studies show that mitochondrial abnormalities involved in the aging process, the occurrence of many diseases (Parkinson’s disease, Alzheimer’s disease, Huntington’s disease, and cancer), and cellular apoptosis all play a very important role; additionally, mitochondria are more closely related to the generation of free radicals. Although oral cancer studies showed that manoalide decreased ΔΨ_m_ [5,10], there was no proof of mitochondrial dysfunction including OXPHOS respiration, OXPHOS protein, and dynamic changes. We are the first to discover that manoalide increases mitochondrial fission protein and lowers OXPHOS respiration, OXPHOS complex I–V protein, and mitochondrial fusion protein.

## 5. Conclusions

**Figure 6:**
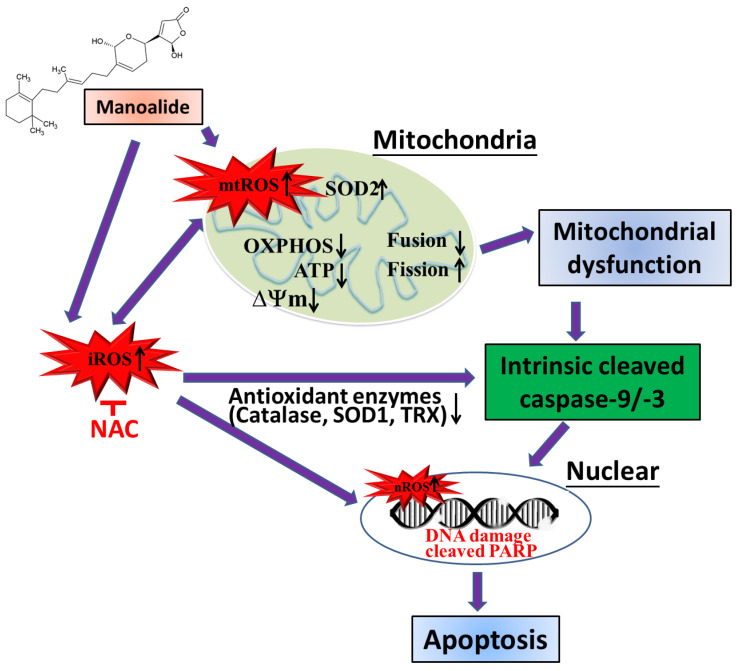
Hypothetical scheme for manoalide-mediated oxidative stress and mitochondrial dysfunction in osteosarcoma cells. mtROS: mitochondrial ROS; iROS: intracellular ROS; nROS: nuclear ROS; SOD2: superoxide dismutase 2, Mn-SOD; mitochondrial manganese superoxide dismutase; SOD1: superoxide dismutase 1, Cu-ZnSOD; TRX: thioredoxin; OXPHOS: oxidative phosphorylation; ATP: adenosine triphosphate; ΔΨm: mitochondrial membrane potential; PARP: poly(ADP-ribose) polymerase; NAC: N-acetylcysteine).

The ROS, mitochondrial malfunction, and mitochondrial (intrinsic) apoptosis pathways of the manoalide-induced apoptosis mechanism in human osteosarcoma MG63 cells are summarized in light of the present findings (Figure 6). Initially, the manoalide-induced overproduction of mitochondrial, intracellular, and nuclear ROS was associated with disrupted antioxidant enzymes (Cu-Zn SOD, catalase, and thioredoxin), whereas increased Mn-SOD antioxidant enzymes led to oxidation stress-damaged cells, nucleus, and mitochondria. On the other hand, manoalide-increased mtROS in MG63 cells led to a decrease in the OXPHOS complex I–V protein of mitochondrial inner membrane bioactivity, ΔΨ_m_, and ATP production and affected the down-regulation of the mitochondrial fusion OPA1 protein and the up-regulation of the mitochondrial fission DRP1 protein, resulting in impaired mitochondrial function. Manoalide-induced cytotoxicity and apoptosis via intrinsic apoptosis proteins activated and cleaved caspases-9/-3 and PARP in OS cells. Adding NAC to reverse the effects of manoalide caused changes in apoptosis pathway proteins, cellular ROS, and antioxidant enzymes. This confirms that oxidative stress is a significant issue in the presence of manoalide. In conclusion, manoalide is a PLA2 inhibitor and shows potential as an innovative alternative treatment in OS, and further advancement of this compound into the preclinical phase is warranted.

## Figures and Tables

**Table 1:** Primary antibody-related information was used in the Western blot analysis in this study.

Antibody	Host Animal	Supplier/Catalogue	Dilution Ratio
**Apoptosis Western Blot Cocktail (pro/p17-caspase-3, cleaved PARP1, muscle actin)**	Rabbit, polyclonal+ monoclonal	Abcam/ab136812	1:250
**Anti-caspase 9**	Rabbit, polyclonal	Cell Signaling/9502	1:1000
**Oxidative Stress Defense (Catalase, SOD1, TRX, smooth muscle Actin) Western Blot Cocktail**	Rabbit, polyclonal	Abcam/ab179843	1:1000
**Anti-SOD2**	Rabbit, monoclonal	Abcam/ab68155	1:1000
**Total OXPHOS Human WB Antibody Cocktail**	Mouse, monoclonal	Abcam/ab110411	1:1000
**Anti-DRP1**	Rabbit, polyclonal	Santa Cruz/SC-32898	1:500
**Anti-OPA1**	Rabbit, polyclonal	Millipore/ABN95	1:1000
**Anti-GADPH**	Rabbit, polyclonal	GeneTex/GTX10018	1:5000

**Table 2:** IC_50_, std. error, R-square, and 95% confidence interval values for MG63 and 143B cells treated with manoalide for 24 and 48 h.

Cell Line	MG63 Cells		143B Cells	
Time (h)	24	48	24	48
IC_50_ (µM)	8.88	8.66	15.07	10.93
Standard error (µM)	1.10	1.12	1.54	1.28
R-square	0.9272	0.9273	0.96	0.96
95% confidence intervals	6.04–11.72	5.79–11.53	11.12–19.01	7.64–14.22

## Supplementary Materials

The following are available online at https://www.mdpi.com/article/10.3390/antiox12071422/s1, Figure S1: The text and results display original and uncropped images of the Western blots for Figure 1G and Figure 2G. Figure S2: The text and results display original and uncropped images of the Western blots for Figure 3G, Figure 4E and Figure 5A,E.
