# Supplementary material for: Manoalide Induces Intrinsic Apoptosis by Oxidative Stress and Mitochondrial Dysfunction in Human Osteosarcoma Cells
Source: Antioxidants (Basel). 2023 Jul 14;12(7):1422. doi: 10.3390/antiox12071422 (PMC10376204; doi:10.3390/antiox12071422)
Supplement: Supplementary file 1 [file antioxidants-12-01422-s001.zip › antioxidants-2471245-supplementary.pdf]

## 1. Supplementary Figures

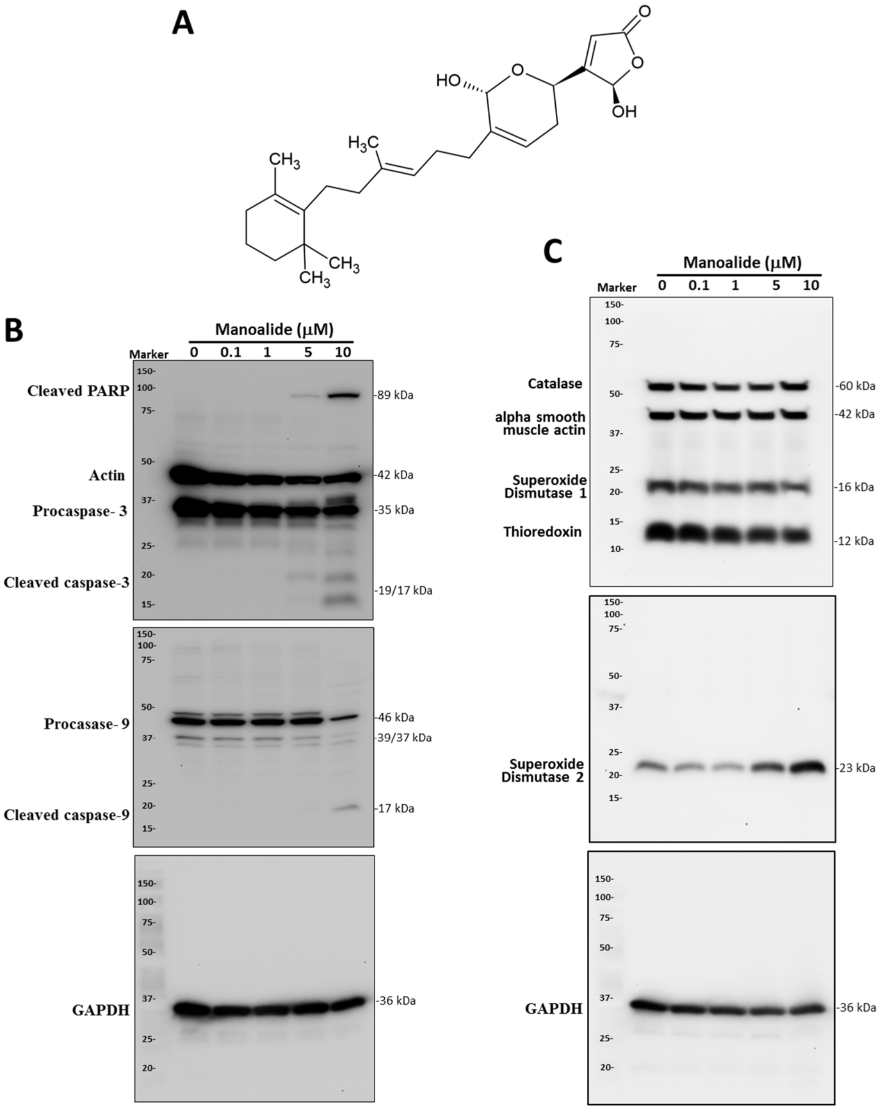


**Supplementary Figure S1.** The text and results display original and uncropped images of the western blots for Figures 1G and 2G. Whole-cell lysate proteins were loaded 20-50 g for western blot utilizing antibodies after treatment with 0, 0.1, 1, 5, and 10 μM of manoalide in MG63 cells for 24 hours. (**A**) Chemical structure of manoalide. (B) The bands of the cleaved caspase-9, procaspase-9, cleaved caspase-3, procaspase-3, cleaved PARP, actin, GADPH, their expected molecular weight, and protein marker molecular weight. (**C**) The bands of catalase, alpha smooth muscle actin, SOD1, thioredoxin, SOD2, and GADPH, their expected molecular weight, and protein marker molecular weight. GADPH, actin, and alpha smooth muscle actin were used as the protein loading controls.

**Supplementary Figure S2.** The text and results display original and uncropped images of the western blots for figures 3G, 4E, 5A, and 5E.

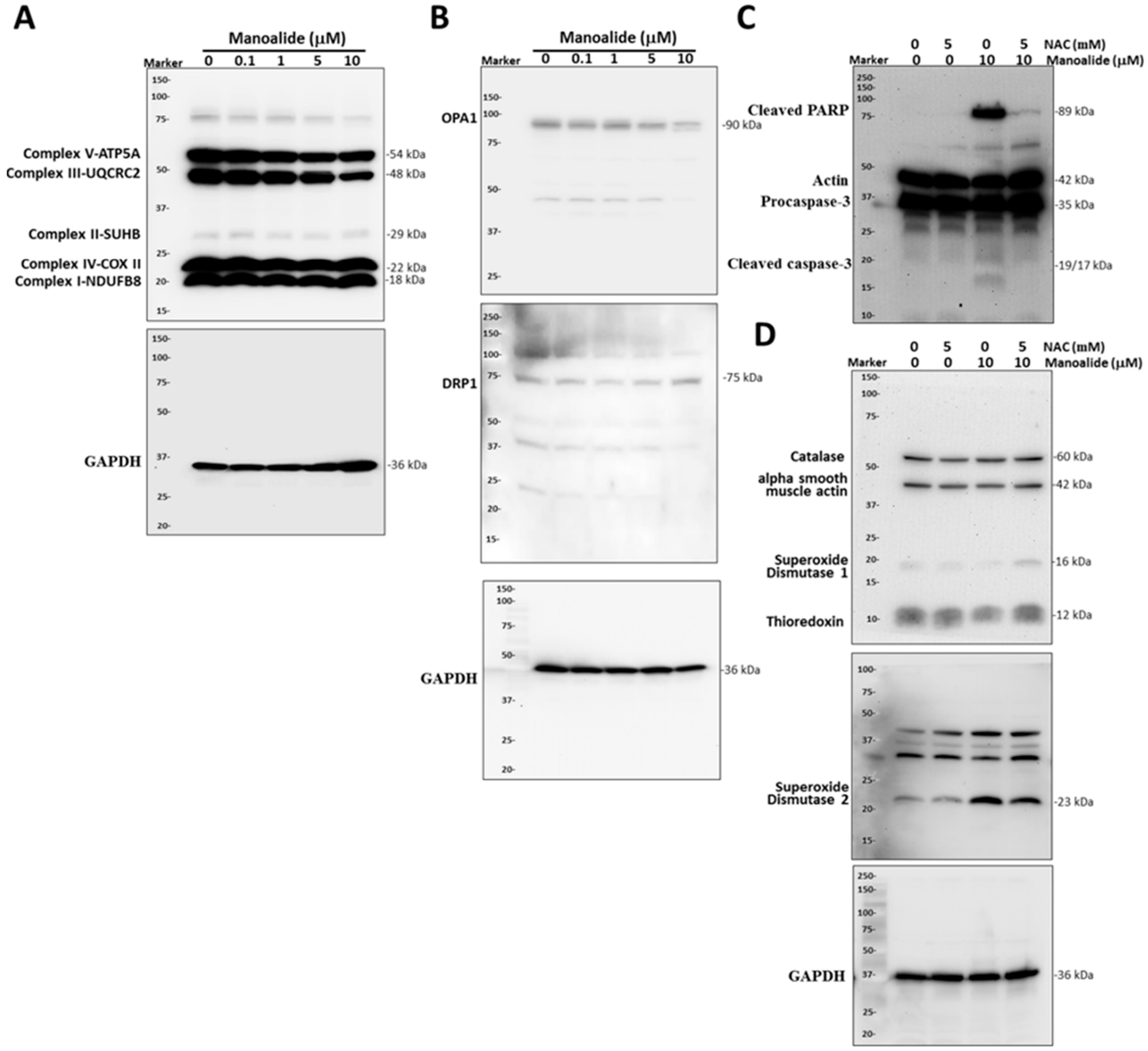


Whole-cell lysate proteins were loaded 20-50 g for western blot utilizing antibodies after treatment with 0, 0.1, 1, 5, and 10 μM of manoalide in MG63 cells for 24 hours. (**A**) The bands of the complex I-NDUFB8, complex II-SUHB, complex III-UQCRC2, complex IV-COX II, complex V-ATP5A, and GADPH, their expected molecular weight, and protein marker molecular weight. (**B**) The bands of OPA1, DRP1, GADPH, their expected molecular weight, and protein marker molecular weight. (C) MG63 cells were pretreated with or without 5 mM NAC for 2 hours to determine the effects of NAC on manoalide-induced apoptosis. The bands of the cleaved caspase-3, procaspase-3, cleaved PARP, actin, their expected molecular weight, and protein marker molecular weight. (D) MG63 cells were pretreated with or

without 5 mM NAC for 2 h to determine the effect of NAC on the action of manoalid-induced oxidative stress defense enzymes. The bands of the catalase, alpha smooth muscle actin, SOD1, thioredoxin, SOD2, GADPH, their expected molecular weight, and protein marker molecular weight. GADPH, actin, and alpha smooth muscle actin were used as the protein loading control.

2. **Supplementary Video**: Graphical abstract animation video of a hypothetical scheme of manoalide-mediated oxidative stress and mitochondrial dysfunction in osteosarcoma cells.
